# Syphilis

**Published:** 1910-11

**Authors:** William Francis Waugh

**Affiliations:** Professor of Therapeutics, Medical Department of Loyola University, (Bennett Medical College), Chicago, Ill.


For Texas Medical Journal.

### Syphilis.

BY WILLIAM FRANCIS WAUGH, A. M., M. D.
Professor of Therapeutics, Medical Department of Loyola University, (Bennett Medical College), Chicago, Ill.

I trust that the fine article on syphilis and its treatment published in the September number of the TEXAS MEDICAL JOURNAL may serve to direct attention to the good work done by my old colleagues in the United States Navy Medical Corps, and by Medical Director Simons in particular. The vital importance of the topic is realized by every one who has studied the sociologic influence of this malady. Dr. Simons has enjoyed unusual opportunities for the study of this malady, and the article before us shows how well these have been utilized. The death of a second member of the faculty with which I am connected, from cerebral syphilis, rapidly following an infection received in the line of duty, in professional attendance, serves to emphasize this.

To the brief historic sketch given, we may add the curious item that Ashmead, who has studied the Peruvian records and traditions, attributes the first appearance of syphilis in man to an infection received during sexual intercourse with the domesticated llama.

Dr. Simons' suggestion as to the peril of lay prescribing are well put. The fact that "mercury is good for syphilis," like "digitalis for heart disease," is an apt illustration of the maxim that "a little knowledge is a dangerous thing." The one great virtue of potassium iodide is not that of curing syphilis, which it doesn't, but that far less harm can be wrought by its unskillful use.

With Dr. Simons' use of calomel, gr. $\frac{1}{2}$, thrice daily in milk sugar trituration for the early stages, I heartily concur. The addition of ammonium chloride to the iodide, to run the mercury out of the body if too active, is an old navy tradition whose value I have many times proved. "Dutch calomel" seems to be forgotten on shore, but the navy surgeon has unusual facilities for studying the effects of treatment, in hospital or on shipboard, and this most powerful of absorbents retains its popularity in the service.

Further commendable points in this paper are the recommendation to abstain from tobacco and alcohol, keeping the alimentary canal clean, avoiding all food that might induce indigestion and the early treatment of the malady. Now that the detection of the treponemia affords a means of earlier diagnosis, we are enabled to eliminate those cases where syphilis was guessed at, and mercury poured in lavishly, with disastrous results when the antagonistic power of the disease was wanting and the destructive metal was left unopposed.

I desire to call especial attention to another phase of syphilis, one we see rather often in cases that have not been treated by the effective method described by Simons. I refer to those in which the infection declares itself in the brain, eye or nose, and swiftly progresses to destruction irreparable of those important structures. For it must not be forgotten that when we talk of curing syphilis, we simply mean we arrest the progress of the malady. No known drug can restore the continuity of torn nerve fibers, or bring back life to a dead eye or necrosed vomer. Hence in attacks of these structures it is of supreme moment that our therapeutics be swift and powerful.

Like the rest, I for many years employed the classic formula—corrosive sublimate and potassium iodide in syrup of sarsaparilla. Becoming dissatisfied with this, and also with saturated solution of potassium iodide, I sought for a more effective remedy.

There is one drug, and one only, that cured syphilis, and that is mercury. The most quickly active salt, and the safest is the biniodide; hence this forms the basis of the formula. Of all iodide combinations I have obtained the quickest action from arsenic iodide, and this duad possesses the advantage of a ready and delicate test for beginning toxic activity, because both elements irritate the eyelids.

Both are liable to irritate the stomach; to prevent this and increase the iodine action I add iodoform.

Finally, to increase lymphatic activity and carry off the debris, I employ the potent vegetable resolvent phytolaccin.

The formula, then, consists of mercury biniodide, 0.003 grams; arsenic iodide, 0.001; iodoform and phytolaccin each, 0.03. This dose is administered an hour before each meal, or three times a day. Every one, two or three days another dose is added, until about the time seven are given per diem the patient begins to experience some irritation of the eyelids. The dose is then reduced until this ceases, and thereafter continued, keeping as close as possible to the irritation point without touching it. We thus obtain the desideratum, the dose that will effect the most destructive metamorphous of the diseased structures, without affecting the normal cells of the body.

The results of this treatment may be judged by the following: A specialist in this city referred to the writer a case where the eye was attacked by syphilis, suggesting the use of iodide in heroic doses to limit the destruction. The patient was placed on the combination here suggested. Next week the specialist wrote to me: "What under heaven have you given this man? Never in my life have I seen such improvement in so short a time!"

